# Palliative care for Parkinson’s disease: suggestions from a council of patient and carepartners

**DOI:** 10.1038/s41531-017-0016-2

**Published:** 2017-05-22

**Authors:** Kirk Hall, Malenna Sumrall, Gil Thelen, Benzi M. Kluger, on behalf of the 2015 Parkinson’s Disease Foundation sponsored “Palliative Care and Parkinson’s Disease” Patient Advisory Council

**Affiliations:** 1 Parkinson’s Disease Patient and Advocate, Highlands Ranch, CO USA; 2 Carepartner for Parkinson’s Disease Patient and Advocate, Castle Rock, CO USA; 3 Parkinson’s Disease Patient and Advocate, Tampa, FL USA; 4 Movement Disorders Center, University of Colorado Denver, Aurora, CO USA

## Abstract

In 2015, the Parkinson’s Disease Foundation sponsored the first international meeting on Palliative Care and Parkinson’s disease and the Patient Centered Outcomes Research Institute funded the first comparative effectiveness trial of palliative care for Parkinson’s disease. A council of Parkinson’s disease patients and carepartners was engaged to assist with both projects. This council wrote the following manuscript as an opinion piece addressed to the clinical and research community on how palliative care could be applied to people living with Parkinson’s disease and their families. The council endorses palliative care as an approach to the care of Parkinson’s disease patients and their families that seeks to reduce suffering through spiritual, psychosocial, and medical support. This approach should start at the time of diagnosis, as this is a very challenging time for patients and carepartners; includes better emotional support, educational resources, and closer follow-up than is currently standard; and continue through end-of-life care and bereavement.

Thomas Graboys, M.D., was a beloved Boston cardiologist who struggled for years with Parkinson’s disease (PD)-related dementia. He died with it in 2015. His book, *Life in the Balance: A Physician’s Memoir of Life, Love, and Loss with Parkinson’s Disease and Dementia*, bared his innermost thoughts about what having Parkinson’s and dementia feels and looks like.^1^ As a physician, he believed in sensitive and effective patient care. His life story and clinical philosophy strongly influenced our thinking on PD palliative care.

When the doctor’s verdict is rendered “PD” is a day we patients will never forget. For some, there is a momentary sense of relief that the accumulating symptoms have a cause and name. For others, the reaction is terror, shock, and confusion. We ask, “What does this diagnosis mean for me?”. Few receive information beyond the diagnosis on what PD is, what we can do about it, and what our future holds. Commonly, we leave the doctor’s office on our own with a levodopa prescription and instructions to return in 3 months.

How would Graboys have broken the news of a Parkinson’s diagnosis if he had been a neurologist and not a cardiologist?

He would take the time to explain what PD is, encouragement about available therapies, and information about the importance of exercise and diet. Graboys would tell us that patients who do well with PD do not let it own them. “You don’t have to do this alone”, he would say. Graboys would also explain at a meeting 1 month later how there was an organization we could join with educational seminars, programs for carepartners, and recommendations for physical and other therapists. He would work closely with the organization to see that care provision was modified as needed for each of us. Graboys would write out any medications he recommended and explain what they were for. He would discuss exercise, diet, and other lifestyle changes that would help enhance our life. He would call it the “plan”.

It was this contract between Graboys and the patient that, if adhered to, would reduce stress and increase the chances of a positive outcome. And because the plan was personal, it was more likely to be honored. Just leaving the office with that plan in hand inspired hope because implicit in that plan was the message that there were things the patient could do to take control of his/her illness and enhance his/her chances of living a fairly normal life.

The Graboys’ allegory contains many of the early stage recommendations of our own prescription for PD Palliative Care and is based on the approach Graboys took with his own patients. While such an approach may not fit every physician or patient we hope, it provides some useful examples of patient-centered care for PD.

The Patient Prescription for PD Palliative Care was created by PD advocates Kirk Hall and Gil Thelen based on their personal experiences and personal interaction with other patients and carepartners.^2^ It outlines recommendations for changes or incremental actions to improve patient quality of life. It is not intended to be an indictment of the current system or the dedicated practitioners who operate within it.

We envision a new, improved approach to Palliative Care based on a “three-legged stool” including the patient’s primary care physician and neurologist (leg 1), a PD palliative care team (leg 2), and a PD support entity (leg 3). The “three legs” are meant to provide support for patients, carepartners, and families throughout the PD journey.

## Early stage: diagnosis to 5 years (honeymoon period)

Given confusion and misperceptions about palliative care, we suggest using the term “supportive care” and discussing this concept as PD Life Enhancement, or something similar. Palliative care should provide a comprehensive, coordinated, and consistent approach for the medical and PD support communities designed to maximize quality of life for patients, carepartners, and families starting at diagnosis and to reduce stress for the duration of the disease and bereavement period.^3^

The key points for diagnosis were included in the Graboys allegory. Another important element is sharing informational resources (see Appendix 1 for an example). We recommend scheduling follow-up a month after diagnosis since many patients are “shell-shocked” and unable to absorb much beyond the words PD. This is an opportunity for the doctor to assess how the patient and carepartner are doing, ask if they have reviewed information resources, and answer questions. The potential value of support groups should be discussed. Finally, it is important for the doctor to outline what information to bring for future appointments to make appropriate care decisions.

We recommend an appointment a year after diagnosis to assess the patient’s and carepartner’s “readiness” to be provided with additional informational resources (see Appendix 2). Most people should be ready at that point and some may have already begun this process on their own. If not, we recommend discussing why they are not ready. Some patients take the “what I don’t know won’t hurt me” approach. It is important to share that in general, patients and carepartners who do best in managing PD take “ownership” of it so that they can properly advocate for themselves and make good choices. We recommend participation in self-efficacy or chronic disease management education programs. This is also a good time to revisit the potential benefits of joining a support group. The doctor should have a working relationship with regional and local support groups.

We propose that at some point in the first couple years following diagnosis, the person with parkinson's (PWP) and carepartner should be asked what they know about palliative care. If they have attended a self-efficacy program, they may know a great deal. Make sure that they understand how it works and the benefits of such a program, emphasizing the need to get involved prior to late stage symptoms in order to avoid unnecessary stress and confusion.

## Middle stage: 5 years to advent of symptoms that substantially affect daily living

The middle stage is a crucial time for patients, carepartners, and families. It is a time when learning can take place relative to late stage. Plans and decisions can be made to make the later stage easier. Wrestling with these issues, including faith, can create acceptance and peace of mind, making the last stage of the journey far less stressful. Tasks should include:

- A personal plan for taking ownership of possible outcomes, including the possibility of financial challenges.
- Develop end of life wish list and legal documents including advance directives.
- Discuss with doctor what his/her role will be in end stage.
- Discuss carepartner plan for assistance and self-care.
- Begin assessing need for in-home safety and for equipment.
- Consider counseling to address faith/spiritual issues or concerns.

Many patients, carepartners, and families miss this extremely important opportunity for a variety of reasons. They may not have as much warning as they think before they are in the thick of late stage and end up scurrying around to find resources, fighting among themselves at a time when they need to be focused on caring for each other. They may not want to face the inevitable decline of their loved one and the difficult decisions this entails, so they take the “ostrich” approach by sticking their heads in the sand. PD palliative care clinics may have value in helping families in this stage.

## Late stage: advent of significant disability/hospice to death/bereavement

In our model, the late stage becomes a matter of implementing plans and preferences identified in the middle stage including hospice when appropriate. Legal paperwork will be available to minimize confusion, misunderstandings, or other “bumps in the road”. Of course, it is not likely that all developments can be foreseen and planned for, but these should be the exception. If the plan includes contingencies based on the nature of specific health issues as they unfold, there can be “course adjustments” as opposed to confusion and stress related to confrontation of unanticipated issues.

We recommended that the patient’s primary neurologist stay engaged with the patient and carepartner in late stage. By that time, a significant relationship based on experience and trust has often been created with both the patient and carepartner. If not, following the Graboys allegory, it should have been. From our perspective it seems that many neurologists are uncomfortable remaining involved after they can no longer “fix” their patient. Training in palliative care or involvement of palliative medicine specialists could help remedy this issue.

It is extremely important to be mindful of carepartner stress/burnout at this stage, and this is an area where a palliative care team could and should add great value. The team needs to be aware that the carepartner can become so overwhelmed that they do not take the time or have the energy to reach out for help. A regular “check-in” should be established that, if missed, would trigger contact by the team. Finally, while bereavement is easy to overlook, we must be mindful of the needs of the carepartner and families following the death of the patient.

### PD support organization proposal

In order for the three-legged stool concept to work consistently and to facilitate development and implementation of programs as well as sharing of best practices, we recommended development of a unified regional program coordinated by a single national entity. Based on our information and experience, we recommended the approach taken by Association of Independent Regional Parkinson Organizations as a model that allows for autonomy and at the same time keeps the benefits of being part of a group, such as timely sharing of information and learning from fellow members’ successes and failures. As a model for a single region, the Muhammad Ali Parkinson’s Center in Phoenix is one potential candidate.

### Medical community proposal

We proposed a fundamental shift in the mindset and training of doctors starting in medical school to facilitate the changes discussed, including getting to know patients and carepartners beyond their medical records and the importance of remaining engaged in late stage to help ensure a “successful transition to death”. We would describe this as one in which the carepartner, family, and medical team can feel at peace because they did everything possible to honor the patient’s wishes about how he/she wanted to die.

This raises an important topic in the minds of the overwhelming majority of PD patients that needs to be resolved. For most of us, it makes no sense to prolong suffering for patients and, in the process, impose huge medical bills on our families by not giving us the choice to die, when no hope of a cure remains. We deserve to have all reasonable choices available to us without risking a stain on our legacies.

Another recommended area of focus for medical schools is the ethical aspects of working with patients who would be better served elsewhere. This is a sensitive subject because it shines a spotlight on doctors who choose to continue treating a patient despite knowing better options exist. We have seen many patients in our support groups receive inappropriate treatments or be incorrectly told there is nothing more to offer by doctors without PD-specific knowledge or skills.

Finally, we add our voice to the many who have called for development of telemedicine and other technologies to increase access to high-quality care in remote/rural areas and for patients with mobility issues.

## Conclusions

Needs and gaps include:

- “Palliative Care” terminology confusion
- Team approach to palliative care
- Reduction of diagnosis angst
- Planning for end stage beginning in middle stage
- Early and better utilization of hospice
- Role of neurologist in late stage
- Patient control of the manner in which they die
- Carepartner/bereavement needs
- Remote area needs

High-priority areas for future research include:

1. Identify barriers and opportunities in the medical community to implement palliative care.
2. Determine the impact of the implementation of the new approach to PD palliative care on PWP, carepartner, and family’s quality of life at each stage of the disease.
3. Determine the effectiveness of new and existing remote area care alternatives to deliver palliative care.
4. Learn from other palliative care approaches (e.g., cancer) that might improve PD palliative care.

## Electronic supplementary material

Appendix 1

Appendix 2
