# Supplementary material for: Palliative care for Parkinson’s disease: suggestions from a council of patient and carepartners
Source: NPJ Parkinsons Dis. 2017 May 22;3:16. doi: 10.1038/s41531-017-0016-2 (PMC5460163; doi:10.1038/s41531-017-0016-2)
Supplement: Supplementary file 1 — Appendix 1 [file 41531_2017_16_MOESM1_ESM.docx]

**Appendix 1: Newly Diagnosed Parkinson’s Education & Resources**

This resource guide has been assembled by an experienced patient/carepartner group and members of the Parkinson’s disease (PD) medical/research community who share a common goal, which is to improve quality of life for people with Parkinson’s (PWPs), carepartners, and their families. A common concern of the newly diagnosed is how to find the information that they want and need. In this brochure are links to resources that we have found helpful to provide basic information regarding Parkinson’s, including young onset. In the future, we hope a comprehensive guide will be available that would allow you to access a wide range of additional information. **If you are unable for any reason to obtain information you seek, there are services (e.g. local support groups, national organizations) that will enable you to speak directly with someone who can help.**

But first, our PWP/carepartner members want to share a few things they have learned that we think are important for you to know:

1. Give yourself some time to “process” your diagnosis. This is a major unanticipated change in your life. It is natural to have some feelings of fear and anxiety, but remember you can take ownership of this process. Yes, your life will be different, but you will be surrounded by many people in support groups, PD organizations, and the medical community who are dedicated to making your life better! Not to mention the support of family and friends (the same people you would support if they were going through something like this). As soon as you are ready:
2. **Your #1 priority** is to be sure you are working with a doctor that has appropriate experience, training, and education for your condition. Do not assume that your doctor, no matter how much you may like him or her, meets this description! Not all neurologists, for example, have movement disorder expertise that will enable them to recognize the subtle symptoms of PD and recommend appropriate medications and/or therapies. If your doctor is not a good fit for you, or even if you are not sure and want a second opinion, we will provide information in our resource guide to help you locate a movement disorder neurologist in your area.
3. **Your #2 priority** is to understand that exercise has been proven to be an effective way for you to improve your condition and how you feel as well as potentially slowing the progression of the disease. It will help you stay positively engaged and fight off the apathy that some of us experience. Work with your doctor to determine what kinds of exercise would be best for you.
4. **Your #3 priority** is to take ownership of your situation by learning about PD and how you can live well with it. This will enable you and your carepartner to take an active role in the management of your condition, including providing information about your symptoms (include all symptoms, whether or not you think they are related), any changes you have experienced, things that concern you, medications you are taking, other conditions you may have, and more. If you have concerns, ask questions! If your doctor consistently does not take the time to answer your questions, find a new one! Your obligation is to yourself and your family!
5. **Your #4 priority** is to locate and join a PD support group. “Test drive” one or two, if necessary, to find one that is comfortable for you and your carepartner. If you have trouble locating a support group, contact your regional support organization for suggestions. Get involved!
6. **If you are in a remote area,** your options may be limited. We know people who have teamed with a local neurologist working in conjunction with a movement disorder specialist that you can visit occasionally. Another option is telemedicine, which allows you to receive care using communication technology. Explore these options with your doctor to find an arrangement that works for you.
7. **Stay engaged!** The steps above will get you moving in a positive direction. With PD there are good days and bad days. Just know during a bad day that the good days will come back. Own each bad day and don’t let it turn into a bad week. You do not have to go through this alone!
8. **There is a need for newly diagnosed patient participation in clinical research!** To learn more visit <https://foxtrialfinder.michaeljfox.org/register/> and complete the profile.

**RESOURCES**

**Help locating a movement disorder neurologist and why this is important**

- Michael J. Fox Foundation (MJFF): [**https://www.partnersinparkinsons.org/find-movement-disorder-specialist?cid=aff_00032**](https://www.partnersinparkinsons.org/find-movement-disorder-specialist?cid=aff_00032)
- **Parkinson Disease Foundation (PDF): http://www.pdf.org/spring12_specialist**

**Exercise information**

- Davis Phinney Foundation (DPF): [**http://www.davisphinneyfoundation.org/living-pd/10tools/?gclid=Cj0KEQjw75yxBRD78uqEnuG-5vcBEiQAQbaxSNfO0tFlTMxBMKAMkKJ6jp6-tzI7Y4nwRBFoEliVcgcaAkdv8P8HAQ**](http://www.davisphinneyfoundation.org/living-pd/10tools/?gclid=Cj0KEQjw75yxBRD78uqEnuG-5vcBEiQAQbaxSNfO0tFlTMxBMKAMkKJ6jp6-tzI7Y4nwRBFoEliVcgcaAkdv8P8HAQ)
- Brian Grant Foundation (BGF): [**http://www.briangrant.org/**](http://www.briangrant.org/)
- National Parkinson Foundation (NPF): [**http://www.parkinson.org/understanding-parkinsons/treatment/Exercise/Neuroprotective-Benefits-of-Exercise**](http://www.parkinson.org/understanding-parkinsons/treatment/Exercise/Neuroprotective-Benefits-of-Exercise)
- Parkinson Disease Foundation (PDF): [**http://www.pdf.org/en/parkinson_exercise_impact**](http://www.pdf.org/en/parkinson_exercise_impact)

**Newly diagnosed information**

- Parkinson Disease Foundation (PDF): [**http://www.pdf.org/symptoms**](http://www.pdf.org/symptoms)
- National Parkinson Foundation (NPF): [**http://www.parkinson.org/understanding-parkinsons/what-is-parkinsons**](http://www.parkinson.org/understanding-parkinsons/what-is-parkinsons)
- Michael J. Fox Foundation (MJFF): [**https://www.michaeljfox.org/understanding-parkinsons/index.html?navid=understanding-pd**](https://www.michaeljfox.org/understanding-parkinsons/index.html?navid=understanding-pd)
- American Parkinson Disease Association (APDA): [**http://www.apdaparkinson.org/parkinsons-disease/understanding-the-basics/**](http://www.apdaparkinson.org/parkinsons-disease/understanding-the-basics/)

**Young onset information**

- APDA: [**http://www.apdaparkinson.org/national-young-onset-center/**](http://www.apdaparkinson.org/national-young-onset-center/)
- NPF: [**http://www.parkinson.org/understanding-parkinsons/what-is-parkinsons/young-onset-parkinsons**](http://www.parkinson.org/understanding-parkinsons/what-is-parkinsons/young-onset-parkinsons)

**Help locating a support group (PWP & carepartner)**

- NPF: [**http://www.parkinson.org/find-help/resources-in-your-community**](http://www.parkinson.org/find-help/resources-in-your-community)
- PDF: [**http://www.pdf.org/en/support_list**](http://www.pdf.org/en/support_list)
- APDA: [**http://www.apdaparkinson.org/resources-support/local-resources/**](http://www.apdaparkinson.org/resources-support/local-resources/)
- PDF: [**http://www.pdf.org/en/airpo**](http://www.pdf.org/en/airpo)

**Help locating a carepartner support group**

- Parkinson’s Health (PH): [**http://www.parkinsonshealth.com/Caring-for-Someone-with-PD/Support-Groups.aspx**](http://www.parkinsonshealth.com/Caring-for-Someone-with-PD/Support-Groups.aspx)

**Talk directly to a person who can help**

- NPF: [**http://www.parkinson.org/find-help/helpline**](http://www.parkinson.org/find-help/helpline)
- PDF: [**http://www.pdf.org/en/ask_expert**](http://www.pdf.org/en/ask_expert)

MJFF: [**https://www.partnersinparkinsons.org/parkinsons-advocate-program?cid=aff_00032**](https://www.partnersinparkinsons.org/parkinsons-advocate-program?cid=aff_00032)
