# Supplementary material for: Palliative care for Parkinson’s disease: suggestions from a council of patient and carepartners
Source: NPJ Parkinsons Dis. 2017 May 22;3:16. doi: 10.1038/s41531-017-0016-2 (PMC5460163; doi:10.1038/s41531-017-0016-2)
Supplement: Supplementary file 2 — Appendix 2 [file 41531_2017_16_MOESM2_ESM.docx]

**Appendix 2: Parkinson’s Education & Resources: 1+ years since diagnosis**

This resource guide has been assembled by an experienced patient/carepartner group and members of the Parkinson’s disease (PD) medical/research community who share a common goal, which is to improve quality of life for people with Parkinson’s (PWPs), carepartners, and their families. We designed it for PWPs and carepartners who are not considered “newly diagnosed” (typically one year since diagnosis).

We want to make it easier for you to find the information and resources that will make it possible for you to be informed and advocate for yourselves effectively. The good news is that, thanks to the dedication of Parkinson resource organizations, just about any information you may need is available on their websites. The problem is that, for many of us, the specific information we seek is not always easy to find within those websites. Others may not be armed with enough information to know what they should be looking for.

This guide will provide links to topics that are important for all “seasoned” PWPs and carepartners to understand. That said, we also want to equip you to find information you seek on almost any topic related to PD. Using your favorite internet search engine (like Google at www.google.com), enter Parkinson’s and _________ (any topic such as fatigue, driving, carepartner issues, depression, anxiety, sleep issues, etc.) in the search box and click “Enter” on your computer keyboard. A list of information resources will appear. Some of the information may not be reliable, so pay attention to the source. Much of the reliable information will be from PD organizations or medical websites such as Parkinson Disease Foundation (PDF), National Parkinson Foundation (NPF), Michael J. Fox Foundation (MJFF), the Mayo Clinic, National Institutes of Health (NIH), and more. Since developments and new information are ongoing, note the date of articles to see which are most current. Another tip to get the most current information is to type in the current year (i.e. 2015) after your subject title in the search box.

But first, we want to share our recommendations and experiences for living well with PD:

- **Continue to make exercise a priority.**
- **Learn about palliative care.** Check out the palliative care links in the resource list below. Attend programs provided in your area that explain palliative care and why it is important.
- **Participate in clinical research trials.** When you do this, you accomplish two things. First, you help with the advancement of knowledge that will lead to a cure. Second, you learn things that may help you. Information on specific studies, including availability, location, and timing of research trials can be found at <https://foxtrialfinder.michaeljfox.org/>.
- **Learn everything you can about PD.** This applies to both PWPs and carepartners. By doing this, you will have a better idea of what to expect in terms of symptoms and progression. Also, it will enable both of you to advocate for yourselves, ask informed questions, and become active/proactive in the management of your health.
- **Prepare for your doctor appointments.** Remember that doctors are very busy individuals who want to provide you with the best care possible. Help make the limited time you have together in appointments productive by preparing a list that includes your current prescriptions, supplements, and symptoms; observations/information regarding your condition; and a list of questions regarding your condition, symptoms, treatment, medications, alternative therapies, or new developments you have heard about that may apply to you.
- **If you are not comfortable with your doctor for any reason, talk to him or her about it.** If you are unable to resolve problems that are important to you, find another doctor! Your number-one obligation is to yourself and your carepartner.
- **Set meaningful goals and work to accomplish them.** If this has always been your approach, continue it. If it has not, resolve to start. Hold yourself accountable and ask your carepartner to do the same.
- **Stay in touch with your passions.** Some of the non-motor problems associated with PD can include depression, anxiety, and apathy. You may be able to reduce these kinds of issues by engaging in activities that have been important to you in the past.
- **Communicate with each other.** It is crucial to keep the lines of communication between you and your carepartner open. Tell each other what you are thinking and feeling. Share the things you are worried about and problem-solve together.
- **Attempt to “live in the moment” as much as possible.** Learn from the past and move on. Plan for the future, but do not dwell on the uncertainty that it surely contains.
- **Balance.** Your “PD life” takes place in the context of your overall life. It will be beneficial for both of you to keep the two integrated and balanced as much as possible.
- **Perspective.** Continue to find the joy in your lives. Celebrate the small victories. Do NOT let PD own you!
- **Take care of yourself (Carepartners)** Ask for help. Solicit assistance as needed from family members and/or friends. Make time for yourself. Stay engaged with your passions. Attend to your personal wellness.
- **Patience (Carepartners)** PD mood swings and/or cognitive problems can be very hard on relationships. No matter how good your communication, it is likely that your partner will sometimes act or react in ways that are not tactful or appropriate. Try very hard not to take these things personally. At a later time, communicate about what happened if you can.

**RESOURCES**

**Apathy/importance of staying engaged**

- [**http://movementdisorders.ufhealth.org/2013/11/26/apathy-and-parkinsons-disease/**](http://movementdisorders.ufhealth.org/2013/11/26/apathy-and-parkinsons-disease/)

**Carepartner wellness**

- [**https://www.michaeljfox.org/foundation/news-detail.php?parkinson-disease-information-for-carepartners-and-loved-ones&smcid=ag-a30U00000004i07&s_src=adwords&s_subsrc=adwords_caregiving_blogpost&smcid=ag-a30U00000004i07&gclid=Cj0KEQiA4LCyBRCY0N7Oy-mSgNIBEiQAyg39thb6-0J405edhulKbtDTD-9bX599AzU0L0n1jFj2jiwaAsLK8P8HAQ**](https://www.michaeljfox.org/foundation/news-detail.php?parkinson-disease-information-for-caregivers-and-loved-ones&smcid=ag-a30U00000004i07&s_src=adwords&s_subsrc=adwords_caregiving_blogpost&smcid=ag-a30U00000004i07&gclid=Cj0KEQiA4LCyBRCY0N7Oy-mSgNIBEiQAyg39thb6-0J405edhulKbtDTD-9bX599AzU0L0n1jFj2jiwaAsLK8P8HAQ)
- [**http://www.pdf.org/en/caregiving_fam_issues?gclid=Cj0KEQiA4LCyBRCY0N7Oy-mSgNIBEiQAyg39ttgyKIq8S-bGBTZxzchsp6Se_32dkxELj7Mqx972qMcaAoJb8P8HAQ**](http://www.pdf.org/en/caregiving_fam_issues?gclid=Cj0KEQiA4LCyBRCY0N7Oy-mSgNIBEiQAyg39ttgyKIq8S-bGBTZxzchsp6Se_32dkxELj7Mqx972qMcaAoJb8P8HAQ)
- [**http://www.pdf.org/en/coping_skills_parkinson_carepartner**](http://www.pdf.org/en/coping_skills_parkinson_carepartner)
- **http://journals.lww.com/neurologynow/Fulltext/2015/11030/Anger_Management__Many_neurologic_conditions_can.14.aspx**

**Deep brain stimulation**

- [**http://www.parkinson.org/understanding-parkinsons/treatment/surgery-treatment-options/Deep-Brain-Stimulation**](http://www.parkinson.org/understanding-parkinsons/treatment/surgery-treatment-options/Deep-Brain-Stimulation)
- [**http://www.ninds.nih.gov/disorders/deep_brain_stimulation/deep_brain_stimulation.htm**](http://www.ninds.nih.gov/disorders/deep_brain_stimulation/deep_brain_stimulation.htm)
- [**http://www.pdf.org/en/surgical_treatments?gclid=Cj0KEQiA4LCyBRCY0N7Oy-mSgNIBEiQAyg39tgjM4e6gh53Rgp0sSALEdyZy7T22Tj4eRL_baPidUFsaAnIQ8P8HAQ**](http://www.pdf.org/en/surgical_treatments?gclid=Cj0KEQiA4LCyBRCY0N7Oy-mSgNIBEiQAyg39tgjM4e6gh53Rgp0sSALEdyZy7T22Tj4eRL_baPidUFsaAnIQ8P8HAQ)

**Depression/Anxiety**

- [**https://www.michaeljfox.org/understanding-parkinsons/living-with-pd/topic.php?emotions-depression**](https://www.michaeljfox.org/understanding-parkinsons/living-with-pd/topic.php?emotions-depression)
- [**http://www.pdf.org/en/combating_depression**](http://www.pdf.org/en/combating_depression)
- [**http://www.parkinson.org/understanding-parkinsons/non-motor-symptoms/anxiety/What-are-the-Treatment-Options-for-Anxiety**](http://www.parkinson.org/understanding-parkinsons/non-motor-symptoms/anxiety/What-are-the-Treatment-Options-for-Anxiety)

**Driving**

- [**http://www.pdf.org/parkinson_briefing_driving?gclid=Cj0KEQiA4LCyBRCY0N7Oy-mSgNIBEiQAyg39trxo7KzWfjTzg26fvtgXt5OWnDaLAS2NTBjjVVag9asaAlUh8P8HAQ**](http://www.pdf.org/parkinson_briefing_driving?gclid=Cj0KEQiA4LCyBRCY0N7Oy-mSgNIBEiQAyg39trxo7KzWfjTzg26fvtgXt5OWnDaLAS2NTBjjVVag9asaAlUh8P8HAQ)
- [**http://www.parkinson.org/understanding-parkinsons/living-well/activities-of-daily-living/driving-with-pd**](http://www.parkinson.org/understanding-parkinsons/living-well/activities-of-daily-living/driving-with-pd)

**Dyskinesia**

- [**https://www.michaeljfox.org/understanding-parkinsons/living-with-pd/topic.php?dyskinesia&smcid=ag-a30U00000004hVt&gclid=Cj0KEQiA4LCyBRCY0N7Oy-mSgNIBEiQAyg39thoaSYHh-rXNhafs6Rjo10B1966_I7nn3LXESTKIb70aAme28P8HAQ**](https://www.michaeljfox.org/understanding-parkinsons/living-with-pd/topic.php?dyskinesia&smcid=ag-a30U00000004hVt&gclid=Cj0KEQiA4LCyBRCY0N7Oy-mSgNIBEiQAyg39thoaSYHh-rXNhafs6Rjo10B1966_I7nn3LXESTKIb70aAme28P8HAQ)

**End of life issues/planning**

- [**http://hospiceandlifecarecenter.org/reso/begin-conversation/?gclid=Cj0KEQiA4LCyBRCY0N7Oy-mSgNIBEiQAyg39tnISsba05cM1DCA5N27C-z7pBC5_hWxQSyxaO9I7PZ4aAkMG8P8HAQ**](http://hospiceandlifecarecenter.org/reso/begin-conversation/?gclid=Cj0KEQiA4LCyBRCY0N7Oy-mSgNIBEiQAyg39tnISsba05cM1DCA5N27C-z7pBC5_hWxQSyxaO9I7PZ4aAkMG8P8HAQ)

**Fatigue/chronic tiredness**

- [**http://www.parkinson.org/sites/default/files/fatigue-and-parkinsons.pdf**](http://www.parkinson.org/sites/default/files/fatigue-and-parkinsons.pdf)

**Gastrointestinal issues**

- [**http://www.pdf.org/en/gastrointestinal_problems_pd**](http://www.pdf.org/en/gastrointestinal_problems_pd)

**Living well with PD**

- [**http://www.davisphinneyfoundation.org/living-pd/webinar/videos/10-commandments/**](http://www.davisphinneyfoundation.org/living-pd/webinar/videos/10-commandments/)
- [**http://www.davisphinneyfoundation.org/living-pd/**](http://www.davisphinneyfoundation.org/living-pd/)

**Medications/Treatments**

- [**http://www.pdf.org/en/meds_treatments**](http://www.pdf.org/en/meds_treatments)
- [**http://www.mayoclinic.org/diseases-conditions/parkinsons-disease/basics/treatment/con-20028488**](http://www.mayoclinic.org/diseases-conditions/parkinsons-disease/basics/treatment/con-20028488)

**Melanoma**

- [**https://www.michaeljfox.org/foundation/news-detail.php?parkinson-disease-linked-to-melanoma**](https://www.michaeljfox.org/foundation/news-detail.php?parkinson-disease-linked-to-melanoma)

**Motor symptoms**

- [**http://www.pdf.org/symptoms_primary**](http://www.pdf.org/symptoms_primary)
- [**http://www.parkinson.org/Understanding-Parkinsons/Motor-Symptoms**](http://www.parkinson.org/Understanding-Parkinsons/Motor-Symptoms)

**Non-motor symptoms**

- [**http://www.pdf.org/symptoms_nonmotor_early**](http://www.pdf.org/symptoms_nonmotor_early)
- [**http://www.apdaparkinson.org/parkinsons-disease-non-motor-symptoms/**](http://www.apdaparkinson.org/parkinsons-disease-non-motor-symptoms/)

**Pain**

- [**http://www.pdf.org/en/pain_pd**](http://www.pdf.org/en/pain_pd)

**Palliative care/hospice**

- [**http://www.parkinsonsresource.org/carepartner/quality-of-life-palliative-and-hospice-care/**](http://www.parkinsonsresource.org/caregiver/quality-of-life-palliative-and-hospice-care/)
- [**https://getpalliativecare.org/whatis/disease-types/parkinsons-disease-palliative-care/**](https://getpalliativecare.org/whatis/disease-types/parkinsons-disease-palliative-care/)

**Parkinson’s dementia**

- [**http://www.alz.org/dementia/parkinsons-disease-symptoms.asp**](http://www.alz.org/dementia/parkinsons-disease-symptoms.asp)
- [**https://www.nia.nih.gov/alzheimers/publication/lewy-body-dementia/basics-lewy-body-dementia**](https://www.nia.nih.gov/alzheimers/publication/lewy-body-dementia/basics-lewy-body-dementia)

**Parkinson’s psychosis/hallucinations**

- [**http://www.parkinson.org/understanding-parkinsons/non-motor-symptoms/Psychosis/What-are-the-Treatment-Options-for-Psychosis**](http://www.parkinson.org/understanding-parkinsons/non-motor-symptoms/Psychosis/What-are-the-Treatment-Options-for-Psychosis)
- [**http://www.pdpsychosis.com/?%2Bparkinsons+paranoiaPD+PsychosisParanoia&gclid=Cj0KEQiA4LCyBRCY0N7Oy-mSgNIBEiQAyg39tp8Bq7KgBHsW-PNlfSqJYi89a6xqNLKhbmE7XZ15d1gaAuyC8P8HAQ#uti-block11**](http://www.pdpsychosis.com/?%2Bparkinsons+paranoiaPD+PsychosisParanoia&gclid=Cj0KEQiA4LCyBRCY0N7Oy-mSgNIBEiQAyg39tp8Bq7KgBHsW-PNlfSqJYi89a6xqNLKhbmE7XZ15d1gaAuyC8P8HAQ#uti-block11)

**REM Behavior Disorder**

- **http://www.healthline.com/health/sleep/rem-sleep-disorder#Overview1**

**Restless leg syndrome**

- [**http://www.apdaparkinson.org/restless-leg-syndrome-and-parkinsons-disease/**](http://www.apdaparkinson.org/restless-leg-syndrome-and-parkinsons-disease/)

**Sleep issues**

- [**https://sleepfoundation.org/sleep-topics/parkinsons-disease-and-sleep**](https://sleepfoundation.org/sleep-topics/parkinsons-disease-and-sleep)
- [**http://www.apdaparkinson.org/parkinsons-and-the-night/**](http://www.apdaparkinson.org/parkinsons-and-the-night/)
- [**http://www.pdf.org/en/sleep_disturbance**](http://www.pdf.org/en/sleep_disturbance)

**Talk directly to a person who can help**

- NPF: [**http://www.parkinson.org/find-help/helpline**](http://www.parkinson.org/find-help/helpline)
- PDF: [**http://www.pdf.org/en/ask_expert**](http://www.pdf.org/en/ask_expert)
- MJFF: [**https://www.partnersinparkinsons.org/parkinsons-advocate-program?cid=aff_00032**](https://www.partnersinparkinsons.org/parkinsons-advocate-program?cid=aff_00032)
